# Point Siamese Network for Person Tracking Using 3D Point Clouds

**DOI:** 10.3390/s20010143

**Published:** 2019-12-24

**Authors:** Yubo Cui, Zheng Fang, Sifan Zhou

**Affiliations:** Faculty of Robot Science and Engineering, Northeastern University, Shenyang 110819, China

**Keywords:** 3D person tracking, convolutional neural networks, LIDAR

## Abstract

Person tracking is an important issue in both computer vision and robotics. However, most existing person tracking methods using 3D point cloud are based on the Bayesian Filtering framework which are not robust in challenging scenes. In contrast with the filtering methods, in this paper, we propose a neural network to cope with person tracking using only 3D point cloud, named Point Siamese Network (PSN). PSN consists of two input branches named template and search, respectively. After finding the target person (by reading the label or using a detector), we get the inputs of the two branches and create feature spaces for them using feature extraction network. Meanwhile, a similarity map based on the feature space is proposed between them. We can obtain the target person from the map. Furthermore, we add an attention module to the template branch to guide feature extraction. To evaluate the performance of the proposed method, we compare it with the Unscented Kalman Filter (UKF) on 3 custom labeled challenging scenes and the KITTI dataset. The experimental results show that the proposed method performs better than UKF in robustness and accuracy and has a real-time speed. In addition, we publicly release our collected dataset and the labeled sequences to the research community.

## 1. Introduction

Person tracking is a key issue in both computer vision and mobile robotics. For example, for person following robots, they usually need to know the position of target person to follow. Therefore, person tracking is necessary for these robots. Currently, most person tracking approaches are based on visual information [1,2,3]. Cameras have many advantages for tracking problems, such as they are compact and cheap, and they can also provide abundant information. However, visual tracking also has its own limitations in practice, especially for autonomous driving car and target following robot. For example, visual tracking is usually sensitive to illumination change which autonomous car and following robot always suffer. Second, visual tracking usually provide pixel coordinate of the tracking target, but lacks accurate target distance information which is usually important for obstacle avoidance or path following control.

In addition to visual sensors, presently 3D laser scanners are also widely used in mobile robots. Compared to 2D images, 3D point cloud generated by laser scanners has more accurate distance measurement and is more robust against illumination change. For this reason, 3D point cloud is also widely used for object or person tracking. However, in contrast to 2D image processing, the processing of point cloud data has its own challenges. First, point cloud data is unordered. For example, a point cloud containing N points has N! permutations to represent, and different representation leads to different feature extraction, which makes it especially hard for neural networks to learn point cloud features end-to-end. Second, nowadays point cloud generated by 3D laser scanners is usually much sparser than 2D image, especially at long distances. Sparse point cloud carries little environmental information compared to dense 2D image, which makes it difficult to extract features from point cloud while feature extraction is usually a key step in the tracking problem. Furthermore, compared with rigid objects tracking (such as cars), person tracking using sparse point cloud is more challenging since it is more difficult to extract stable features with the non-rigid characteristics.

To deal with the tracking problem using point cloud, previous works usually use filtering methods, such as [4,5,6,7,8]. Those methods are usually composed of two interleaved steps: (i) Target detection: classify point cloud clusters based on handcrafted features [9] and (ii) Target tracking: determine the most likely cluster that may be the target person by the filtering-based tracker [5,6,8]. Generally, those methods can successfully track a person in most common places in real time. However, the separation of detection and tracking not only increases the computational burden, but also introduces new challenges to the tracking problem. For example, those methods usually have to classify point cloud clusters for every frame using the target detector. Meanwhile, the trackers have to take the output of the detector as their input, which means they cannot track a person if the detector fails to detect. Unfortunately, target detection failure is common in practice, especially in challenging scenes such as those containing fast rotation or occlusion.

**Figure 1:**
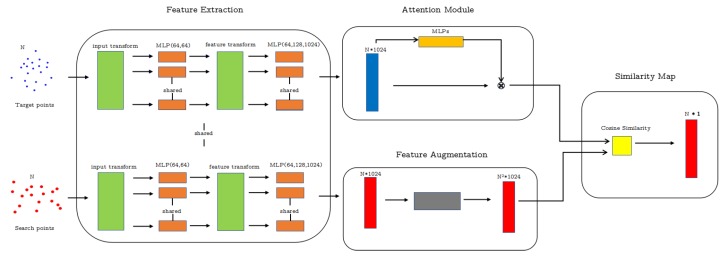
The architecture of the proposed PSN. The top branch inputs the target point cloud in the first frame from the detector (only use once for initialization) as a template point cloud and the search branch inputs all point clouds in the search box during tracking. An attention module in the template branch outputs a weight for each feature in the template point cloud. The network outputs a score map describing the similarity of the two points clouds. We use [16] as the backbone network to extract features.

Recently, deep learning has shown powerful performance in processing point cloud tasks [10,11,12,13,14], and there are many new methods to handle point cloud data [10,15,16]. Currently, there are some learning-based methods that could extract more robust features from point cloud than traditional methods. In this paper, we demonstrate that we can successfully perform person tracking using point clouds by a deep learning method. Inspired by the 2D image tracking method [2], we propose a neural network for point cloud tracking based on similarity computation, named Point Siamese Network (PSN), as shown in Figure 1. We take advantage of deep learning to create feature space for point clouds and propose a similarity map to track a person. The similarity map is calculated based on the feature space of the template and search point clouds. The point, which has the highest score in the similarity map, has the highest similarity with the point cloud of the target person. Additionally, we apply an attention module to weight the features of the template point cloud, thus in different scenarios, the network could flexibly extract features. We evaluate the proposed network both quantitatively and qualitatively with respect to accuracy and robustness. The experimental results show that the proposed method is capable of tracking a person using 3D point cloud in real time.

To summarize, the main contributions of this paper are as follows:

- We propose an end-to-end network to handle person tracking only using point cloud, which has higher robustness than UKF. The proposed network only uses 3D point cloud to predict the point in the search cloud that is most similar to the target person, therefore it does not depend on the front-end person detector during tracking.
- We propose a score map based on pseudo-cloud to compare similarities between different points. In addition, for further performance improvement, we apply an attention module to weight the features of the template point cloud to select features with the most useful information.
- We release our training dataset (a point cloud dataset with labeled person) and testing sequences (three challenging point cloud sequences) to the research community. The dataset and sequences are useful for the research community since it is difficult to find such challenging person tracking scenarios on the Internet for public access.

## 2. Related Work

In this section, we provide a brief summary of existing object tracking methods that use 2D images and 3D point clouds.

### 2.1. Object Tracking Using 2D Images

Early works in visual tracking were mainly based on Correlation Filters (CF). Bolme et al. [17] introduced CF to object tracking and achieved high speed, however, their accuracy is not enough. Henriques et al. [18] added HOG features based on [17] and has a better performance. Bertinetto et al. [1] combined two features to improve the accuracy and robustness of tracker. However, they are both sensitive to scale changing. Danelljan et al. [19] solved this by using 33 different scales. After that, Danelljan et al. [20,21] used deep features and an interpolation model which highly improved the performance of the tracker. However, these methods are usually accurate and fast for rigid object tracking, but for non-rigid object tracking like people or animals, those methods struggle to handle the fast deformation of target object because CF method is a template-like method.

After having great success in many computer vision tasks, deep learning began to show power in object tracking. For example, Qi et al. [22] proposed a tree-like nets to deal with different stages in tracking and took a weighted sum of all nets to track object finally. Held et al. [23] treated object tracking as a regression of target movement and achieved very good real-time tracking performance. However, the network fails to track when the target object moves fast. Recently, Bertinetto et al. [2] proposed a fully-convolution siamese network to track target objects. They solved object tracking by using a similarity computation between the first-frame image and subsequent frames. Li et al. [3] added RPN [24] to the siamese to get a higher tracking accuracy than CF-based methods. After that, Zhu et al. [25] improved the training technique and the discriminating ability of network. Li et al. [26] modified the network and made it deeper; they also proposed a new cross-correlation layer and achieved state-of-the-art performance.

### 2.2. Object Tracking Using 3D Point Clouds

Previous works about 3D object tracking were usually based on filter methods and could be divided into two parts depending on whether the object is rigid. Different from rigid objects, non-rigid objects are deformable and may change shape during tracking. Therefore, for non-rigid object tracking, especially for person tracking, early works usually depend on trained classifier [9,27,28] to detect objects even if their shape have changed. After getting detection results from the detector every frame, they usually use filter methods [5,6] to match the detection results, which can track objects. Therefore, the difference between rigid and non-rigid object tracking usually lies in the front-end detector. In these filter-based tracking methods, they usually need the front-end detector to provide the position of objects, and the filters use these detection results to match frame to frame. Therefore, the filters do not have the ability to predict the position of objects. Take the UKF tracker for example, it consists of prediction and update. After getting detection results from a front-end detector, UKF computes a Kalman gain which is used to track object by updating state. These methods can track object in most common scenes. However, they heavily rely on the front-end object detector, which easily results in failure in dynamic scenes with strong disturbances, such as fast rotation.

Recently, with the development of point cloud processing using deep neural network [10,15,16,29], object tracking using CNN becomes popular. For example, Luo et al. [30] convert LiDAR data to Bird Eye Views (BEV) to detect objects, and use the overlap between detection from current and past/future predictions to track objects. However, the overlap relies on the accuracy of the current detection, therefore the tracker is easily affected by the detector’s performance. Giancola [31] is the first one who uses the siamese network to find the best candidate to track. However, they also need a search method to provide search candidates which are input into their network. Davi et al. [14] and Martin et al. [32] also proposed deep neural network to detect and track objects. However, they both need RGB information from camera to get an accurate detection result. Thus, they are sensitive to illumination change. Additionally, the methods mentioned above still rely on a detector or search method to provide a priori information. Different from these works, in this paper, we propose an end-to-end network to track a person directly and do not depend on a detector.

## 3. Method

In this work, we deal with the tracking problem as a similarity issue between the template cloud and the search cloud, which are the inputs to the two branches. The template cloud is the point cloud of the target person from first frame person detector, and the search cloud is the cloud in an area where the target person may appear. In our method, we aim to find the target person in the search cloud by using template cloud as a reference. In our algorithm, we only detect the person using SVM [33] in the first point cloud frame to get the target person to track, and consider the detection result as the template cloud for the person. Meanwhile, we also use the detection result to define a search box for the current point cloud frame and get the search cloud. After the first frame of initialization, we do not use the detector during tracking until there is no point in the search box. The two point clouds will be sent to our proposed network PSN to find the maximum response point in the current search point cloud corresponding to the template point cloud. Finally, the PSN outputs a similarity score map describing the similarity between each point in the current search point cloud and the template point cloud. The higher score indicates the higher similarity, thus it also implies that it has found the target person if it found the highest score point. After obtaining the target person, we update the search box and could get a new search point cloud, therefore we could form a closed loop for tracking directly. Because our tracker has the ability to find where the target is, we do not depend on the detection results to track. We will now introduce all modules in detail.

### 3.1. Person Detection

Different from previous point cloud tracking algorithms which detect the person in each frame, we only detect person in the first frame to initialize the target person to be tracked and do not detect during tracking unless our tracker has no input search cloud. Here, we use SVM [33] and handcrafted features to detect person in the first point cloud frame. We first cluster the point cloud into many cloud clusters based on [34], then we use SVM to detect the person at the first frame based on the slice features proposed by [9,35], the feature will be saved and the output cluster is then sent to the template branch of the proposed network. We then freeze the detector until the tracker has no input search cloud. We also calculate the centre point of this template point cloud and define a search box based on the centre point. The center point is also the centre of the search box. The size of the search box is fixed during tracking. With this search box, we can get the search point cloud, which are to be fed into the search branch of the proposed network, in the current point cloud frame. Here, we use notations *Z* and *X* to denote the template point cloud and the search point cloud respectively.

### 3.2. Point Siamese Network

As shown in Figure 1, our proposed method consists of two branches that share parameters to extract features so that the two branches are encoded by the same transformation. We first resample the template point cloud *Z* and search point cloud *X* to the same size *N*, and then pass them through a feed-forward feature extraction network inspired by [16] to learn local features. This feature extraction network produces size *N × F* feature matrices for both template branch and search branch. In addition, we design an attention module in the template branch to generate weight for each template feature. The template features are multiplied by the weights before calculating the similarity map. We use the weighted template features and the search features to obtain the final similarity map which is used to track the target person. We describe the four parts shown in Figure 1 in detail in the following sub-sections.

#### 3.2.1. Feature Extraction

Feature extraction is the most important step for the tracking problem. Nowadays, there are some excellent feature extraction algorithms based on deep neural network [15,29,36]. However, many networks are too slow for real-time tracking. To achieve fast tracking capability, the network should have a skillful architecture so that it has lower computation cost. In addition, for accurate tracking, it is better to directly input the point cloud into the network instead of converting the point cloud into other format to avoid losing too much information. For these reasons, we use a PointNet [16] layer to extract features from the point cloud.

PointNet layer learns a spatial encoding of each point. To learn representation which is invariant to transformations, such as rigid transformation, it first aligns all points to a canonical space before feature extraction by a mini-network (T-Net), and then uses another T-Net to align feature space established by the first Multilayer Perceptron (MLP), which are called input transform and feature transform respectively. Finally, it uses MLPs to extract high-level features. In our work, the layers just extract local features rather than global features of the input point cloud. Therefore, the features represent only local point features but not the whole point cloud, which are more useful in computing the similarity map between points.

#### 3.2.2. Feature Augmentation

**Figure 2:**
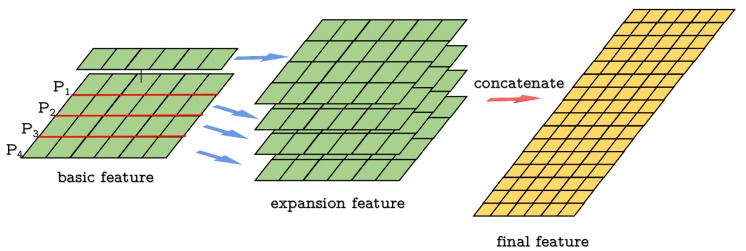
Feature expansion example. For a 4 × 6 feature, we repeat each point feature to the number of the points, which is 4 in this example and get four 4 × 6 point features. We concatenate these features and get the final 16 × 6 feature.

After feature extraction in search branch, we obtain *N × F* size feature, where *N* is the number of points in the point cloud and *F* is the number of channels. We first take every 1 × F row of the search feature which represents the feature of point and repeat it *N* times. Therefore, the 1 × F feature becomes *N × F* for each point in *X*. We concatenate all these repeated features to get a new search feature. The concatenated search feature becomes *N${}^{2}$× F* size. Each new *N × F* feature in the concatenated search feature represents a point in *X* rather than a point cloud. We call this augmented point **pseudo-cloud**, which is directly used in similarity computation, and the pseudo-cloud consists of *N* same points. An example of the augmentation operation is shown in Figure 2. In Figure 2, every feature represents the feature of every point and the expansion feature represents the feature of pseudo-cloud.

#### 3.2.3. Attention Module

Different from the augmentation in search branch, we do another weighted operation in the template branch before the similarity computation.

Usually, different spatial points play different roles in the tracking. In order to filter noise and use high-dimensional features flexibly, we design a spatial attention module in the template branch. The module is only added in template branch, because we believe that the template branch plays a guiding role comparing to search branch during tracking. After extracting template features, the template branch sends these features to the attention module to generate a weight for each spatial point. The original features multiply with these weights to obtain weighted features.

**Figure 3:**
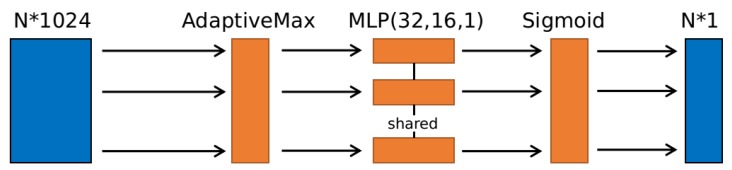
The Attention module: The AdaptiveMax pooling layer outputs a size of N × 48 features. Features are then sent to a MLP for dimension reduction, and then the sigmoid function will compute a coefficient.

Extracted features are firstly sent to an AdaptiveMax Pooling layer to be resized to a fixed size, three MLP layers are followed to output a coefficient for each spatial point. The coefficient is input to a sigmoid function to obtain the final weight. The attention module is shown in Figure 3.

(1) $$F_{t}^{\prime }=F_{t}\times w$$

where $F_{t}$ is the original features in template branch, and *w* is the final weight for each feature. $F_{t}^{\prime }$ is the final weighted features we used to compute the similarity score map.

#### 3.2.4. Similarity Map

After augmentation in search branch and weighting in template branch, we use the *N${}^{2}$× F* search feature and *N × F* weighted template feature to obtain a similarity map *S*. We calculate the similarity of each pseudo-cloud feature and template cloud feature respectively and get a *N × 1* similarity matrix for each pair finally. For each pair of $\left(PC_{i},Z\right)$, where $PC_{i}$ is a pseudo-cloud in search branch and *Z* is the template point cloud, we obtain a corresponding element $S_{i,j}$ in *S* by computing their cosine similarity:

(2) $$S\left(i,j\right)=CosSim\left(F_{pc(i,j)},F_{j}^{\prime }\right)$$

where $F_{pc(i,j)}$ and $F_{j}^{\prime }$ are corresponding deep features for *j*-th point in the *i*-th pseudo-cloud $PC_{i}$ and *j*-th point in template cloud *Z*.

Therefore, each element $S_{i,j}$ in *S* represents the similarity between a point in pseudo-cloud $PC_{i}$ and a point in template point cloud *Z*, and each row $S_{i}$ represents the similarity between pseudo-cloud $PC_{i}$ and template point cloud *Z*. To have a clearer representation and reduce the computational cost, we convert $S_{N,N}$ to $S_{N}$ as follows:

(3) $$S_{i}=Max\left(S_{i}\right)+Min\left(S_{i}\right)$$

**Figure 4:**
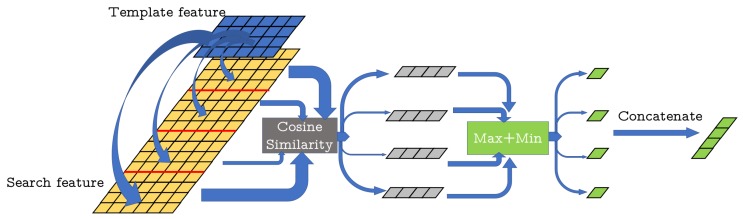
Cosine Similarity Computation. For 4 × 6 template feature and 16 × 6 search feature, we compute cosine similarity of pseudo-cloud feature and template feature and could get a 1 × 4 similarity matrix. For each matrix we take the sum of its maximum and minimum values as the similarity of the point. The final 4 × 1 similarity map are concatenated from all summed value of pseudo-cloud in *X*.

The operation reduces the dimension of *S* from *N × N* to *N × 1* and obtains a single value to describe the similarity for each pseudo-cloud to template cloud. And since the pseudo-cloud is the multiple copies of each point in the search cloud *X*, the obtained value actually describes the similarity between a point in the search cloud *X* and the template cloud *Z*. This makes it easier to decide which point in the search point cloud is the target point we need to track. The whole process of calculating similarity is shown in Figure 4.

Additionally, we try to replace Equation (3) by computing the mean of $S_{i}$:

(4) $$S_{i}=Average\left(Sum\left(S_{i}\right)\right)$$

Equations (3) and (4) have different meaning in choosing points to represent. Because a value is computed by a point in template cloud, different values represent different points in template cloud. The former one believes that Max and Min points are much more important than the other points, but the later one takes all points into consideration. To verify the effect of this step, we compare these two different operations in our experiments.

Finally, we obtain a *N × 1* size similarity map describing similarity score between all points in the search point cloud *X* and the template point cloud *Z*. If a point is much more similar to template cloud *Z*, it will have a higher score in the map. We use this character to decide which point is the target point.

The proposed network is symmetric and fully convolutional, thus the size of the input point clouds could be arbitrary, and the computation is more efficient and less burdensome.

### 3.3. Training Network

Training input pairs are two different person point clouds, and a point in the search point cloud *X* is considered to be belonging to a positive example if it is within radius *R* of the centre:

(5) $$q\left(x\right)=\left\{\begin{array}{ccc} 1 & \; & d_{x}<R\\ 0 & \; & otherwise \end{array}\right.$$

The reason for this kind of labeling is that it is natural to consider that the target person is in the centre of the search point cloud. In addition, the search box is formed by the center of the template point cloud, and the center point is also the center of search box. Thus, the points adjacent to the centre are more likely to belong to the target person in the search box.

We train our network with positive and negative pairs and adopt the binary cross-entropy loss:

(6) l(p,q)=-qlogp-(1-q)log(1-p))

where *p* is the similarity score of a single point $P_{i}$ in the search point cloud *X*, *q* is its ground-truth label ∈{1,0}. Similar to [2], the proposed network also outputs a map of scores per pair. For each search point cloud *X*, the loss of its score map is the mean of the individual losses for each point $P_{i}$∈ *X*:

(7) $$L\left(p,q\right)=\frac{1}{N}\sum_{i=1}^{N}l\left(p_{i},q_{i}\right)$$

## 4. Experiments and Analysis

In this section, we compare the performance of the proposed methods with UKF, a successful method which has been proven in most 3D tracking tasks. A front-end detector described in Section 3.1 is used to initialize the target person for our method at the first frame and input detection results to UKF tracker in every frame. We conducted our experiments on a custom-collected challenging 3D person tracking sequence and the tracking benchmark of KITTI dataset [37].

### 4.1. Implementation Details

In our tracking implementation, we initialize the template point cloud at the first frame by the detector described in Section 3.1. After inputting the template point cloud *Z* and obtaining the search box, PSN starts to track. Meanwhile, because there is no need to extract features in template branch, we can obtain a faster running speed if we freeze template branch. Therefore, unlike other filter-based trackers, the template point cloud will not update during tracking after getting the first frame detection. After getting the search box, we input all point clouds in the search box to PSN to generate the similarity map, and a cosine window is added to the map to penalize large displacements. Once we find the highest score point in the score map, which is also the most similar point with template point cloud in the whole search point cloud *X*, the target box could be obtained, and the search box based on the target box will be updated simultaneously. Therefore, a new search point cloud could be obtained and a same process is used to keep tracking. When there is no point in the search box, we enlarge the box and activate the detector to detect in the enlarged search box. We use the new features obtained by detector to match the saved feature in the first frame to find the target person. The tracking process is described in Algorithm 1.

In the implementation of feature extraction network, a simplified PointNet layer is applied. The first T-Net is 3×64 for raw point cloud data and the second T-Net is 64×64 for first extracted features. The MLPs are 3×64, 64×128, 128×1024 in the feature extraction stage. In the attention module, an AdaptiveMaxPool layer with size 48 is first used to have a fixed feature size, and MLPs in the attention module are 48×32, 32×16, 16×1, which are followed by a sigmoid layer to output the final weight.

To train the proposed network, we use the optimizer from [38] with an initial learning rate 0.002, batch size 5 and training epoch 30. The learning rate is divided by 2 every 10 epochs. *N* is set to 200. All training processes are implemented with a Nvidia Tesla K40C GPU and Pytorch.
**Algorithm 1:** Tracking Process
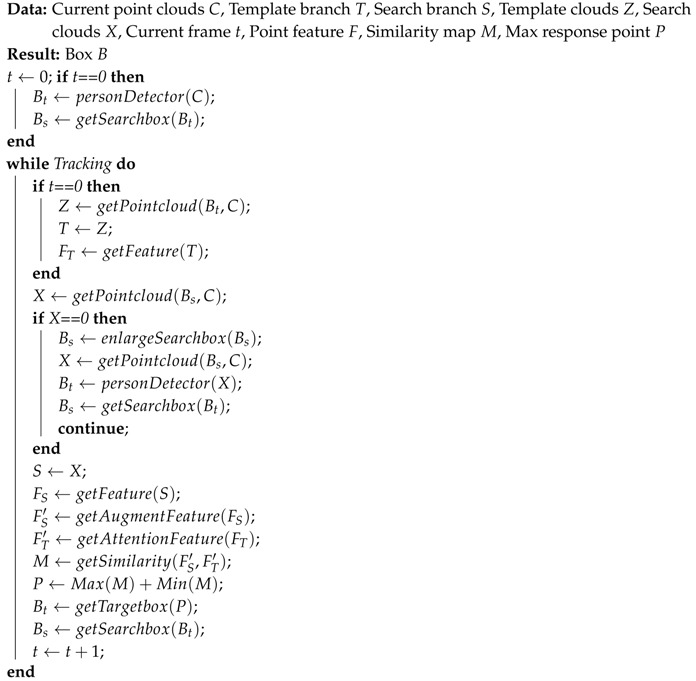


### 4.2. Experiments Setup

**Figure 5:**
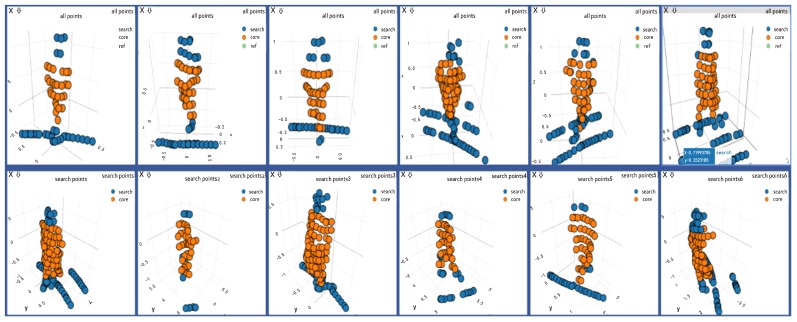
An example of the template and search point clouds for training. The points in yellow are the points whose labels are positive and the points in blue are negative.

We first collected a dataset to train PSN and then used three different sequences to evaluate. The dataset is collected with Velodyne VLP-16 3D LiDAR from different distances, and the training and validation sets contain 8800 frames from 20 different scenes where are more open areas. The frequency of the Velodyne is 10 HZ. The collected dataset provides abundant information for person representation from sparser 3D LiDAR data, such examples of 3D point cloud data are not commonly found in the community. Thus, our network could have a strong robustness to track the person after training. We released our datasets (https://drive.google.com/open?id=1ywJoQjah1lb2iqXOYw31XSnM3Olm4Q7E) to the research community. An example of the dataset can be seen in Figure 5. During training, the template point cloud and the search point cloud are randomly selected and at most 20 frames apart. The labeled sequences include challenging scenes which are not addressed by most of the current methods. Table 1 shows details for these sequences. These sequences (https://drive.google.com/open?id=1cFsBKwSuFqIJSmPuC4qID8RwGNG3HL9y) are also publicly available.

We also trained and evaluated the proposed method on the training set of KITTI [37] tracking benchmark. Sequences 0–16 were used for training, 17 for validation and 19 for testing in our person tracking experiments. (Sequence 20 has no pedestrian to track). The sequence 19 has 62 pedestrians for 3D person tracking. To train the proposed network, we first read and expand the true 3D bounding box of the tracked object from the label, and then extract the point cloud of the object from the expanded bounding box. The extracted point clouds are input to the proposed network to train the model.

In the tracking experiment, we implemented the proposed method using the Robot Operating System(ROS) [39]. The implementation was carried out with Ubuntu16.04 LTS and ROS Kinetic with Intel Core i7-7700HQ CPU and 8GB memory. All coordinates are based on our Velodyne coordinate system. Our experimental video can be found at https://youtu.be/Ul1l-QrjFvs.

### 4.3. Evaluation Metrics

We use three metrics to evaluate the experiments: the accuracy defined by [40], the mean distance error to the box centre introduced by [41], the two metrics are used to evaluate the accuracy of methods. We also define a metric to evaluate the robustness as follows:

(8) $$R=\frac{\sum_{i=1}^{N}K}{NM}$$

where *K* means the number of successful frames in the *i*-th experiment, *N* means how many times of tests on a sequence, *M* is the total length of the test sequence.

To further explore the attention module’s effect in promoting the tracking performance, and the operation in converting $S_{N,N}$ to $S_{N}$, we also compared PSN without the attention module and PSN with mean operation as Equation (4), denoted by PSN-base and PSN-mean respectively.

### 4.4. Experiment Result on Our Sequences

**Figure 6:**
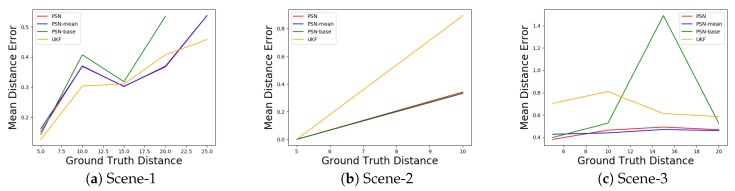
Mean distance error for box centre in the 3 scenes. The X-axis represents the range of distance from the box centre to the coordinate origin. The distances of Scene-2 are all between 5 and 10 thus only have one point for each line, we added (5, 0) to discern easily.

**Figure 7:**
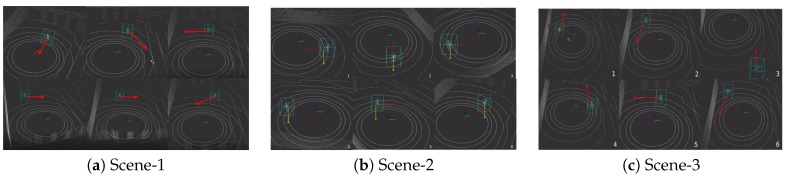
Experiment results on our sequence. The red arrows are the heading direction of the target person, and the yellow one is the X axis of the coordinate system.

The results for accuracy and robustness are reported in Table 2 while mean distance error is reported in Figure 6, the tracking results are shown in Figure 7. We bolded the best data in each column for easy viewing. As Table 2 shows, the proposed three PSN methods outperform UKF both in terms of accuracy and robustness. Moreover, PSN-mean is comparable to PSN in all scenes, and PSN methods with the attention module, including PSN and PSN-mean, are better than PSN-base as we discussed above, especially with respect to robustness performance. This verifies that the robustness of tracking could be improved by adding attention module. Meanwhile, PSN, PSN-mean and PSN-base achieves 14.64 FPS, 14.47 FPS and 14.76 FPS respectively. Therefore, our proposed methods can track the target person at real-time speeds.

In the three tracking scenes considered, the UKF method has much lower robustness in all three scenes. We speculate that this is due to the dependence on the front-end detector, which mostly effects the performance of the tracker. The detector fails to detect a person, especially in Scene-2 and Scene-3, where the target and the Velodyne have large disturbances. Once the detector fails, the tracker is invalid because there is no point cloud input. In contrast, the proposed methods have stronger robustness. We believe that there are two reasons for this. Firstly, the proposed method does not depend on any detector and only inputs the point clouds in the search box. Secondly, the attention module extracts features flexibly which acts as a noise filter when there is a large disturbance, which also contributes to the improvement of accuracy and robustness. The improvement is obvious especially in Scene-2 and Scene-3 in Table 2. Meanwhile, as Table 2 shows, the proposed PSN methods also have higher accuracy than UKF in all three sequences. We speculate that this is because UKF updates its model each time and does not filter noise well. In addition, the novel similarity score map using high-dimensional features could have a better performance than UKF when searching for a similar target.

The mean error distance to the centre of the box is shown in Figure 6. In two out of three scenes, the proposed PSN methods have smaller error than UKF. Figure 6c highlights the importance of the attention module. The networks with an attention module have consistently smaller error even with large disturbances. This also proves that the attention module could filter noise. We also noticed that UKF outperforms the proposed methods in Figure 6a. We speculate that this is due to the sparsity of the input point clouds, thus the network has fewer useful features to use.

### 4.5. Experiments Result on KITTI

**Figure 8:**
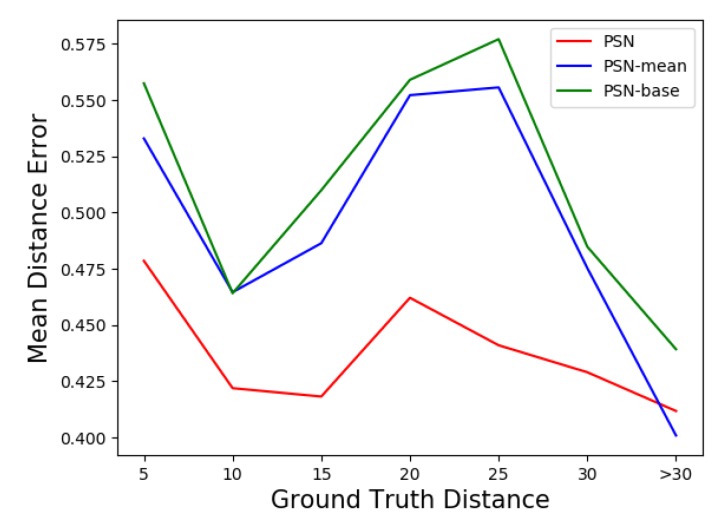
Mean distance error for box centre in KITTI.

**Figure 9:**
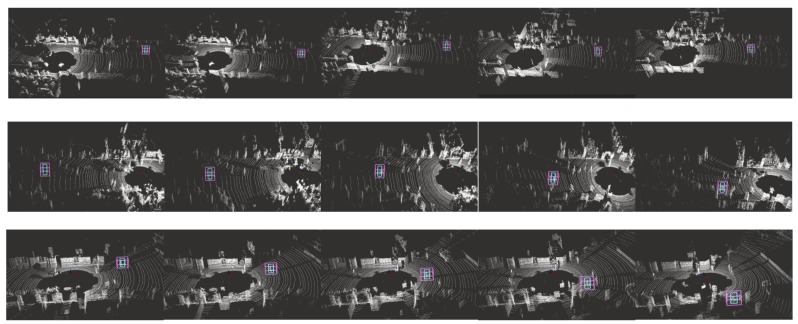
Experiment results on KITTI. Each row shows the one tracking process. The bigger purple box is the search box and the smaller blue one is the target box.

The results of accuracy and robustness on KITTI are reported in Table 3 while mean distance error is reported in Figure 8. In our experiments, the UKF tracker fails to track the person in most cases since the front-end person detector fails, thus we do not have enough statistical data to report the performance of UKF tracker. The KITTI tracking results are shown in Figure 9.

In all the three reported trackers, which do not depend on the front-end person detector, PSN achieves the highest accuracy and highest robustness similar to the result in the previous experiment, while PSN-mean is slightly inferior to PSN. This illustrates that for the tracker with attention module, the tracking performance is possible to degrade if all points are considered during the tracking. Meanwhile, it also shows that the trackers with the attention module have better performance than that without the module. The mean distance error shown in Figure 8 also illustrates the same results. They both show that the attention module could improve the performance of the method. Meanwhile, improper operation in converting similarity map may result in degeneration of the most similar point prediction.

As the two experiment results show, PSN-mean has a little worse performance to PSN. Meanwhile, PSN converges fast in our training process, while PSN-mean takes much more time to train. This also proves that the operation (Equation (3)) of obtaining the final score map *S* is more reasonable.

## 5. Conclusions

In this paper, we proposed a novel end-to-end deep neural network to track a person using only a 3D point cloud. After getting the target person, the proposed network only uses 3D point clouds to predict the point in the search cloud that is the most similar to the point cloud of the target person, therefore it does not depend on the front-end person detector during tracking. We showed that the proposed approach has good performance for person tracking especially in robustness, which is very important for tracking. We also showed that deep neural network could deal with person tracking without detector. Our training datasets and evaluation sequences are all publicly available. In future work, we plan to extend PSN to multiple people tracking.

## Figures and Tables

**Table 1:** Details of evaluation sequences.

Sequence	Scene	Frames
1	Long Distance	772
2	Fast Rotation	497
3	Fast Movement	685

**Table 2:** Details of evaluation results.

	Sequence 1		Sequence 2		Sequence 3	
Method	Acc	Robustness	Acc	Robustness	Acc	Robustness
UKF	31.45%	64.51%	9.35%	11.07%	13.51%	26.27%
PSN	**32.12**%	97.15%	**27.94**%	**96.98%**	20.36%	**86.13%**
PSN-mean	32.08%	**98.96%**	26.88%	84.51%	**20.59**%	75.62%
PSN-base	31.74%	31.22%	27.63%	**96.98%**	18.16%	57.96%

**Table 3:** Details of evaluation results on KITTI.

Method	Acc	Robustness
UKF	fail	fail
PSN	**26.37**%	**77.53**%
PSN-mean	23.23%	67.59%
PSN-base	22.64%	51.11%
